# Venous and arterial thrombosis following extracorporeal photopheresis: A descriptive cohort study using a federated electronic health record network

**DOI:** 10.1111/trf.70300

**Published:** 2026-06-18

**Authors:** Caitlin Raymond

**Affiliations:** ^1^ Department of Pathology and Laboratory Medicine University of Wisconsin–Madison Madison Wisconsin USA

**Keywords:** extracorporeal photopheresis, graft‐versus‐host disease, patient safety, TriNetX, venous thromboembolism

## Abstract

**Background:**

In 2018, the FDA reported VTE events in patients undergoing ECP, but these reports lacked a population‐level denominator to contextualize the risk against the known baseline thrombotic burden of the underlying conditions.

**Study Design and Methods:**

We conducted a descriptive retrospective cohort study using the TriNetX federated EHR network. Patients undergoing ECP with no thrombosis codes in the preceding 6 months or 1 year were identified. The primary outcome was new VTE within 5 and 30 days of the index procedure. Secondary analyses examined VTE subtypes and stratified risk by indication (GVHD vs. CTCL).

**Results:**

Among 3006 patients (1‐year thrombosis exclusion), the 30‐day VTE risk was 6.0% (95% CI, 5.2%–6.9%) and the 5‐day risk was 1.4% (95% CI, 1.0%–1.8%); estimates were consistent across thrombosis exclusion periods. Kaplan–Meier analysis showed gradual event accumulation without periprocedural clustering. VTE risk at 30 days was markedly higher in GVHD patients (8.1%; 95% CI, 6.8%–9.5%) than CTCL patients (3.3%; 95% CI, 2.1%–5.0%). Among GVHD patients, upper extremity DVT, PE, and lower extremity DVT contributed approximately equally. Arterial thrombotic events were infrequent (1.5% at 30 days).

**Conclusion:**

VTE following ECP occurs at a rate consistent with baseline thrombotic risk in the underlying populations and does not cluster periprocedurally, strongly suggesting that patient‐level factors rather than the procedure drive the observed risk. A small contributory procedural effect cannot be excluded without a controlled comparator study.

**Abbreviations**
8‐MOP: 8‐methoxypsoralen
CPT: current procedural terminology
CTCL: cutaneous T‐cell lymphoma
DVT: deep vein thrombosis
ECP: extracorporeal photopheresis
EHR: electronic health record
FDA: US Food and Drug Administration
GVHD: graft‐versus‐host disease
HSCT: hematopoietic stem cell transplantation; NOS, not otherwise specified
ICD‐10‐CM: International Classification of Diseases, Tenth Revision, Clinical Modification
ICD‐10‐PCS: International Classification of Diseases, Tenth Revision, Procedure Coding System
MAUDE: manufacturer and user facility device experience
PE: pulmonary embolism
UVA: ultraviolet A
VTE: venous thromboembolism

## 1 INTRODUCTION

Extracorporeal photopheresis (ECP) is a treatment modality in which buffy coat elements are removed from the circulation via an apheresis device, treated with the psoralen photosensitizer methoxsalen, exposed to ultraviolet A (UVA) light, and reinfused to the patient.^1^ Originally developed for the treatment of cutaneous T‐cell lymphoma (CTCL), ECP has since been applied to a range of immune‐mediated conditions, including graft‐versus‐host disease (GVHD), cellular‐mediated cardiac transplant rejection, and chronic lung allograft dysfunction.^2^, ^3^

ECP is generally considered to have an excellent safety profile compared to systemic immunosuppressive therapies. Reported adverse effects are typically mild, including fever, photosensitivity, volume shifts, and electrolyte disturbances.^4^ However, in 2018 the US Food and Drug Administration (FDA) issued a safety communication to healthcare providers reporting cases of venous thromboembolism (VTE), including pulmonary embolism (PE) and deep vein thrombosis (DVT), in patients undergoing ECP with the CELLEX Photopheresis System.^5^ The FDA described seven PE events and two DVT events, largely in patients receiving ECP for GVHD, with a mean time from treatment to event of approximately 1.2 days.

Subsequent analysis of the FDA's Manufacturer and User Facility Device Experience (MAUDE) database by Jacobs et al. identified 43 clinically significant thrombotic events associated with ECP, approximately four times the number reported in the original FDA communication.^6^ These events occurred across multiple indications, including lung allograft rejection and CTCL, although a significant number occurred in GVHD. The timing of thrombosis was reported for 17 of the 43 cases, with the median time from procedure to thrombosis being 1 day.

Outside of these regulatory reports, the published ECP literature contains minimal evidence of thrombotic complications despite over three decades of clinical use and hundreds of thousands of procedures performed. Isolated case reports have described central venous thrombosis in a patient undergoing ECP via port catheter^7^ and one episode of central venous line‐associated thrombosis among 385 pediatric ECP procedures.^8^ A Japanese multicenter study reported a single serious adverse event of thrombosis in the device itself during treatment.^9^ Notably, laboratory studies examining the effects of 8‐methoxypsoralen (8‐MOP) and UVA on platelets have found that while platelet activation markers (CD62P, CD63) are upregulated after 8‐MOP/UVA exposure, this does not translate into platelet aggregation or clinically significant changes in soluble P‐selectin or platelet factor 4.^10^, ^11^ Coagulation parameters have been shown to remain stable during ECP treatment, with no significant changes observed even after 12 months of therapy.^12^ When thrombotic events have been reported in the ECP literature, they appear primarily related to central venous catheter access rather than to a systemic prothrombotic effect of the procedure.^4^, ^13^, ^14^

Despite these reports, the fundamental challenge in interpreting this safety signal remains the absence of a population‐level denominator. The total number of ECP procedures and patients treated annually is unknown, and the conditions for which ECP is used—particularly GVHD—carry a substantial baseline risk of thrombosis independent of any procedural exposure.^15^, ^16^, ^17^, ^18^, ^19^, ^20^ Without population‐based incidence estimates in ECP‐treated patients, it is not possible to determine whether VTE risk is attributable to ECP itself or to the underlying disease.

The purpose of this descriptive study was to estimate the incidence of venous and arterial thrombosis following ECP using a large, federated electronic health record (EHR) network, to characterize the temporal distribution of events, and to compare VTE risk across a subset of ECP indications.

## 2 MATERIALS AND METHODS

### 2.1 Ethics

This study used de‐identified data accessed through the TriNetX federated research network. Because all data are de‐identified in compliance with the Health Insurance Portability and Accountability Act (HIPAA) and accessed through a federated architecture in which patient‐level data do not leave the contributing healthcare organizations, this study was exempt from institutional review board approval and the requirement for informed consent under 45 CFR 46.104(d) (4).

### 2.2 Data source

The data used in this study were accessed on March 5, 2026, from the TriNetX US Collaborative Network (TriNetX LLC, Cambridge, MA, USA),^21^ a federated research network providing access to de‐identified electronic medical records (diagnoses, procedures, medications, laboratory values) from approximately 70 healthcare organizations.

### 2.3 Study design

This was a descriptive retrospective cohort study. The aim was to characterize the incidence and temporal distribution of thrombotic events in patients who underwent ECP, not to establish a causal relationship between ECP and thrombosis. Accordingly, no comparator group was included. A comparator cohort (e.g., GVHD patients not receiving ECP) would address a different question—namely, whether ECP independently increases thrombotic risk beyond the baseline risk of the underlying disease. While that is an important question, it would require adjustment for the many confounders that differ between ECP‐treated and non‐ECP‐treated patients (e.g., disease severity, central venous catheter use, immunosuppressive regimens, functional status), most of which are not reliably captured in claims‐level data. The present study instead provides the descriptive epidemiology needed to contextualize the existing safety signal.

### 2.4 Study population

The study cohort included patients who underwent ECP, identified by CPT code 36522 (Photopheresis, extracorporeal) or ICD‐10‐PCS code 6A650ZZ (Phototherapy, Circulatory, Single). To identify new‐onset thrombotic events occurring after ECP, patients were required to have no diagnosis of VTE (ICD‐10‐CM I82), PE (ICD‐10‐CM I26), cerebral infarction (ICD‐10‐CM I63), or acute myocardial infarction (ICD‐10‐CM I21) within a defined exclusion period prior to the index ECP date. The exclusion period was designed to remove patients with recent thrombotic events, thereby reducing diagnostic code carryover from pre‐existing conditions. Patients with a remote history of thrombosis predating the thrombosis exclusion window were retained in the analysis, as these individuals remain at risk for new thrombotic events and their exclusion would not be appropriate for a descriptive incidence estimate.

The initial analysis used a 6‐month exclusion period, yielding a cohort of 3744 patients (3096 included in the analysis). A secondary sensitivity analysis using a 1‐year exclusion period yielded 3633 patients (3006 included in the analysis). In both cases, patients whose index ECP procedure occurred more than 20 years ago were excluded by the TriNetX platform as a data quality constraint; this accounted for the difference between the initial and analyzed cohort sizes. The index event was defined as the first ECP procedure meeting the inclusion criteria.

### 2.5 Subgroup analyses

To evaluate whether VTE risk differed by indication, subgroup analyses were performed using the 1‐year thrombosis exclusion cohort. Patients with GVHD were identified by ICD‐10‐CM codes for chronic GVHD (D89.811), acute GVHD (D89.810), acute on chronic GVHD (D89.812), and complications of bone marrow transplant (T86.09, T86.00), yielding 1744 patients in the analysis. Patients with CTCL were identified by codes for Sézary disease (C84.1), mycosis fungoides (C84.0), and mature T/NK‐cell lymphomas (C84), yielding 631 patients in the analysis. These subgroups represent a rough division of ECP indications based on diagnoses that are robustly captured by administrative coding; they do not represent a complete classification of all indications. Patients receiving ECP for other indications (e.g., solid organ transplant rejection) are included in the all‐indication cohort but were not analyzed separately.

### 2.6 Outcomes

The primary outcome was new VTE, defined as any diagnosis of other venous embolism and thrombosis (ICD‐10‐CM I82) or pulmonary embolism (ICD‐10‐CM I26), occurring within 30 days of the index ECP procedure (day 1 through day 30). A secondary time window of 5 days (day 1 through day 5) was used to evaluate early periprocedural risk. Arterial thrombotic events were defined as cerebral infarction (ICD‐10‐CM I63) or acute myocardial infarction (ICD‐10‐CM I21) within the same time windows. The observation window began on day 1 (the day after the index ECP procedure) rather than day 0 (the day of the procedure) because EHR data record diagnoses at calendar‐day resolution, and events coded on the same day as the index procedure cannot be reliably determined to have occurred after the procedure. A supplemental sensitivity analysis including day 0 was performed to assess whether this analytic choice materially affected the findings (Supplemental Table S1).

VTE subtypes were analyzed separately and included PE (I26), lower extremity DVT (I82.4, I82.401, I82.402, I82.409, I82.41, I82.43, I82.44, I82.49, I82.4Y, I82.4Z), upper extremity DVT (I82.6, I82.61, I82.62), and VTE not otherwise specified (I82.8, I82.89, I82.9, I82.90). These categories are not mutually exclusive; a single patient could contribute events to more than one subtype.

### 2.7 Statistical analysis

Risk was calculated as the proportion of patients experiencing the outcome within the specified time window, with exact (Clopper–Pearson) 95% confidence intervals. Kaplan–Meier survival analysis was used to estimate the probability of remaining event‐free over the observation period, with patients censored after the last recorded fact in their medical record. Analyses were performed within the TriNetX platform, with confidence intervals calculated separately.

### 2.8 Use of artificial intelligence

The author used Claude (Anthropic) to assist with manuscript drafting and formatting. All study design, data analysis, data interpretation, and critical review of the final manuscript were performed solely by the author.

## 3 RESULTS

### 3.1 Study population

Baseline demographic characteristics are presented in Table 1. Across all cohorts, patients were predominantly male (57%–59%) and White (74%–77%). The median age at index was 56 years (IQR 27) in the all‐indication cohorts, 54 years (IQR 27) in the GVHD cohort, and 64 years (IQR 19) in the CTCL cohort, consistent with the older age of onset of CTCL. To contextualize thrombotic risk, the percentage of each cohort on anticoagulants within 10 days of the index ECP was determined. In the 6‐month and 1‐year exclusion cohorts, 31% were exposed to anticoagulation prior to index ECP procedure. In the GVHD cohort, 37% were exposed, with 15% exposed in the CTCL cohort. We note that the TriNetX platform cannot distinguish between therapeutic and prophylactic anticoagulation. Baseline coagulation parameters obtained within 5 days before the index ECP procedure were within normal limits across all cohorts (Table 2), with mean INR values of 1.12–1.19, mean aPTT of 29.7–30.9 s, and mean PT of 13.5–14.1 s.

**TABLE 1:** Baseline demographic characteristics of ECP‐treated patients by cohort.

Characteristic	All indications, 6‐mo exclusion (*N* = 3096)	All indications, 1‐yr exclusion (*N* = 3006)	GVHD (*N* = 1744)	CTCL (*N* = 631)
Age at index, year				
Mean ± SD	51 ± 19	51 ± 19.1	48.6 ± 18.3	61.7 ± 15.8
Median (IQR)	56 (27)	56 (27)	54 (27)	64 (19)
Range	0–90	0–90	1–90	11–90
Sex, *n* (%)				
Male	1752 (57%)	1703 (57%)	1025 (59%)	368 (59%)
Female	1343 (43%)	1303 (43%)	719 (41%)	263 (42%)
Race, *n* (%)				
White	2276 (74%)	2216 (74%)	1345 (77%)	467 (74%)
Black or African American	410 (13%)	388 (13%)	170 (10%)	99 (16%)
Asian	62 (2%)	61 (2%)	38 (2%)	10 (2%)
Ethnicity, *n* (%)				
Hispanic or Latino	209 (7%)	198 (7%)	109 (6%)	22 (3%)
Not Hispanic or Latino	2543 (82%)	2470 (82%)	1440 (83%)	546 (87%)
Anticoagulants, *n* (%) (within 10 days of index ECP)	996 (31%)	916 (31%)	651 (37%)	95 (15%)

*Note*: The 6‐month and 1‐year exclusion cohorts include all ECP indications. The GVHD and CTCL cohorts use the 1‐year thrombosis exclusion. Percentages may not sum to 100% due to missing or unreported data.

Abbreviations: CTCL, cutaneous T‐cell lymphoma; GVHD, graft‐versus‐host disease.

**TABLE 2:** Baseline coagulation parameters within 5 days before index ECP procedure, by cohort.

Parameter	*n*	All, 6‐months	*n*	All, 1‐year	*n*	GVHD	*n*	CTCL
INR, mean ± SD	534	1.15 ± 0.35	506	1.15 ± 0.35	391	1.12 ± 0.22	44	1.19 ± 0.56
aPTT (s), mean ± SD	364	30.9 ± 13.6	350	30.9 ± 13.7	283	29.7 ± 11.5	30	30.4 ± 9.24
PT (s), mean ± SD	513	13.8 ± 3.6	486	13.8 ± 3.57	375	13.5 ± 2.52	44	14.1 ± 5.06

*Note*: Values represent the most recent result within 5 days before the index ECP procedure.

Abbreviations: aPTT, activated partial thromboplastin time; INR, international normalized ratio; n, number of patients with available laboratory data; PT, prothrombin time.

### 3.2 Venous thromboembolism in all ECP patients

Using the 6‐month thrombosis exclusion period, 3096 patients were included in the analysis. The 30‐day risk of any VTE was 6.3% (194/3096; 95% CI, 5.4%–7.2%), and the 5‐day risk was 1.5% (46/3096; 95% CI, 1.1%–2.0%). With the 1‐year thrombosis exclusion period, 3006 patients were analyzed, yielding a 30‐day VTE risk of 6.0% (180/3006; 95% CI, 5.2%–6.9%) and a 5‐day risk of 1.4% (41/3006; 95% CI, 1.0%–1.8%). The consistency of estimates across thrombosis exclusion periods suggests minimal diagnostic code carryover (Table 3).

**TABLE 3:** Risk of venous and arterial thrombosis following extracorporeal photopheresis by thrombosis exclusion period and time window (all indications).

Exclusion period	Window	*N*	Events	Risk (95% CI)	Survival Prob.	Outcome
6 months	5 days	3096	46	1.5% (1.1–2.0)	98.51%	VTE
6 months	30 days	3096	194	6.3% (5.4–7.2)	93.63%	VTE
6 months	5 days	3096	13	0.4% (0.2–0.7)	99.58%	Arterial
6 months	30 days	3096	52	1.7% (1.3–2.2)	98.29%	Arterial
1 year	5 days	3006	41	1.4% (1.0–1.8)	98.63%	VTE
1 year	30 days	3006	180	6.0% (5.2–6.9)	93.91%	VTE
1 year	5 days	3006	11	0.4% (0.2–0.7)	99.62%	Arterial
1 year	30 days	3006	46	1.5% (1.1–2.0)	98.44%	Arterial

*Note*: Confidence intervals are exact (Clopper–Pearson) 95% CIs.

Abbreviations: VTE, venous thromboembolism (ICD‐10‐CM I82 + I26); Arterial, cerebral infarction (I63) or acute myocardial infarction (I21).

**FIGURE 1:**
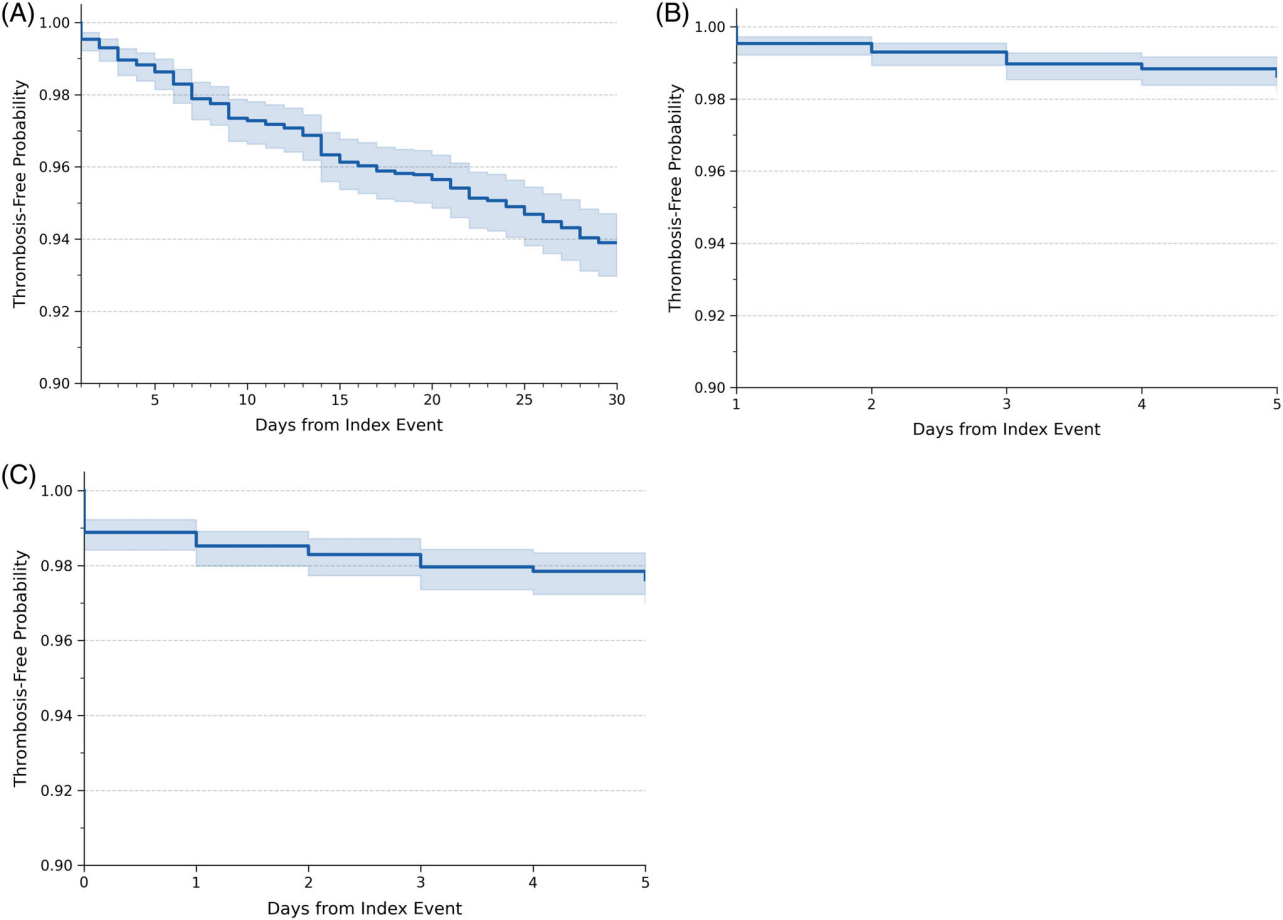
Kaplan–Meier estimates of VTE‐free probability following extracorporeal photopheresis (ECP) in patients across all indications. Kaplan–Meier curves depicting the cumulative thrombosis‐free probability following the index ECP procedure in patients with no history of thrombosis in the preceding year. The outcome was defined as any new diagnosis of venous thrombosis (VTE). Patients were censored at the time of their last recorded clinical encounter. Shaded regions represent 95% confidence intervals. The *y*‐axis was truncated to highlight the shape of the curve near the index ECP procedure. (A) Thrombosis‐free probability over days 1–30 following the index ECP procedure. (B) Thrombosis‐free probability over days 1–5 following the index ECP procedure, excluding events on the day of the procedure. (C) Thrombosis‐free probability over days 0–5 following the index ECP procedure, including events on the day of the procedure.

Kaplan–Meier analysis of the 1‐year thrombosis exclusion cohort demonstrated a gradual, approximately linear decline in VTE‐free survival over the 30‐day observation window, with a survival probability of 93.91% at day 30 (Figure 1A). The 5‐day Kaplan–Meier curve showed minimal event accumulation, with survival remaining above 98.6% throughout the observation period (Figure 1B). There was no evidence of early periprocedural clustering of VTE events.

### 3.3 Arterial thrombotic events

Arterial thrombotic events (cerebral infarction or acute myocardial infarction) were infrequent. Using the 6‐month thrombosis exclusion period, the 30‐day risk was 1.7% (52/3096; 95% CI, 1.3%–2.2%) and the 5‐day risk was 0.4% (13/3096; 95% CI, 0.2%–0.7%). Using the 1‐year thrombosis exclusion period, the 30‐day risk was 1.5% (46/3006; 95% CI, 1.1%–2.0%) and the 5‐day risk was 0.4% (11/3006; 95% CI, 0.2%–0.7%) (Table 3).

### 3.4 Sensitivity analysis: Inclusion of day 0

When the observation window was extended to include day 0 (the day of the index ECP procedure), the 5‐day VTE risk increased from 1.4% to 2.3% (70/3006; 95% CI, 1.8%–2.9%) in the 1‐year thrombosis exclusion cohort and from 1.5% to 2.4% (75/3096; 95% CI, 1.9%–3.0%) in the 6‐month thrombosis exclusion cohort. Arterial thrombotic events similarly increased from 0.4% to 0.6% in both thrombosis exclusion groups. The increase was driven entirely by events coded on the same calendar day as the ECP procedure (day 0), for which temporal ordering relative to the procedure cannot be established in EHR data. These events may represent thromboses that preceded the ECP encounter rather than procedural complications. The inclusion of day 0 increased the 5‐day VTE risk modestly, but the absolute difference was small and attributable to events with uncertain temporal ordering. Importantly, Kaplan Meier analysis of the 0–5 day window also showed gradually accumulation of thrombotic risk, without notable periprocedural clustering (Figure 1C). The overall interpretation of the data—that VTE does not cluster in the immediate periprocedural period—was not materially affected (Supplemental Table S1).

### 3.5 VTE risk by indication

VTE risk at 30 days was markedly higher in patients with GVHD (8.1%; 141/1744; 95% CI, 6.8%–9.5%) compared to patients with CTCL (3.3%; 21/631; 95% CI, 2.1%–5.0%), though the CTCL estimate should be interpreted with caution given the smaller sample size and correspondingly wider confidence interval. In the GVHD cohort, VTE subtypes contributed approximately equally to the overall risk: upper extremity DVT occurred in 2.5% (44/1744; 95% CI, 1.8%–3.4%), PE in 2.3% (40/1744; 95% CI, 1.6%–3.1%), and lower extremity DVT in 2.3% (40/1744; 95% CI, 1.6–3.1%) (Table 4). VTE not otherwise specified accounted for 0.8% (14/1744; 95% CI, 0.4%–1.3%). VTE subtype categories are not mutually exclusive, and a single patient could contribute to more than one category. In the all‐indication cohort, the distribution was similar, with upper extremity DVT (1.8%; 95% CI, 1.4%–2.4%), lower extremity DVT (1.7%; 95% CI, 1.3%–2.2%), PE (1.6%; 95% CI, 1.2%–2.1%), and VTE NOS (0.6%; 95% CI, 0.4%–1.0%) (Table 4).

**TABLE 4:** Thirty‐day risk of venous thromboembolism subtypes following extracorporeal photopheresis, by indication (1‐year thrombosis exclusion).

VTE subtype	All *n*/*N*	Risk (95% CI)	GVHD *n*/*N*	Risk (95% CI)	CTCL *n*/*N*	Risk (95% CI)
All VTE (I82 + I26)	180/3006	6.0% (5.2–6.9)	141/1744	8.1% (6.8–9.5)	21/631	3.3% (2.1–5.0)
PE (I26)	48/3006	1.6% (1.2–2.1)	40/1744	2.3% (1.6–3.1)	—	—
VTE, any (I82)	143/3006	4.8% (4.0–5.6)	111/1744	6.4% (5.3–7.6)	—	—
Lower extremity	51/3006	1.7% (1.3–2.2)	40/1744	2.3% (1.6–3.1)	—	—
Upper extremity	55/3006	1.8% (1.4–2.4)	44/1744	2.5% (1.8–3.4)	—	—
NOS	19/3006	0.6% (0.4–1.0)	14/1744	0.8% (0.4–1.3)	—	—

*Note*: VTE subtype categories are not mutually exclusive; a single patient may contribute to more than one category. Dashes indicate data not available (CTCL subtype analysis not performed due to small sample size). Confidence intervals are exact (Clopper–Pearson) 95% CIs.

Abbreviations: CTCL, cutaneous T‐cell lymphoma; GVHD, graft‐versus‐host disease; NOS, not otherwise specified; PE, pulmonary embolism; VTE, venous thromboembolism.

Kaplan–Meier survival probability at 30 days was 91.81% in GVHD patients and 96.63% in CTCL patients, compared to 93.91% in the overall cohort.

## 4 DISCUSSION

This study provides the first population‐level estimates of thrombotic event incidence following ECP, addressing a critical gap identified by both the 2018 FDA safety communication and subsequent analyses of the MAUDE database.^5^, ^6^ Using a federated EHR network spanning 70 US healthcare organizations, we found that the 30‐day VTE risk following ECP is approximately 6% in the overall ECP population, with the risk concentrated in patients with GVHD (8.1%) compared to CTCL (3.3%). Arterial thrombotic events were infrequent, with a 30‐day risk of 1.5%–1.7%.

These rates are consistent with—and do not appear to exceed—published estimates of VTE incidence in the underlying patient populations. Martens et al. reported that high‐risk allogeneic HSCT recipients, including those with grade 3–4 GVHD, had an incident VTE rate of 10.3% between day 30 and day 100 after transplant.^19^ In a large cohort of 2276 allogeneic HSCT recipients with over 4 years of follow‐up, Kekre et al. found an overall VTE incidence of 8.3%, with both acute and chronic GVHD serving as independent predictors.^18^ El Jurdi et al. reported that the 5‐year cumulative incidence of thromboembolic events in patients with chronic GVHD was 22%, with rates of 38% in patients with severe disease.^22^ Pihusch et al. reported 1‐year VTE rates of 8.1% in allogeneic HSCT recipients with GVHD, compared to 6.8% in those without GVHD and 4.8% in autologous HSCT recipients.^20^ Viewed against this background, the 8.1% 30‐day VTE rate we observed in ECP‐treated GVHD patients falls within the range expected for this population, and the data strongly suggest that the VTE observed after ECP reflects the baseline thrombotic risk of the underlying disease rather than a direct procedural effect.

Perhaps the most clinically important finding is the temporal distribution of VTE events. The 2018 FDA communication described events occurring “during or shortly after” ECP sessions, with a mean time to event of 1.2 days, suggesting a direct periprocedural mechanism.^5^ Our Kaplan–Meier analysis does not support this interpretation. The 5‐day VTE risk was only 1.4%, and the 30‐day survival curve showed a gradual, approximately linear decline without an early inflection point. This pattern is more consistent with background VTE accrual in a medically complex population than with a procedure‐triggered thrombotic event.

The difference in VTE risk between GVHD and CTCL patients further supports this interpretation. Allogeneic transplant recipients with GVHD carry elevated VTE risk due to multiple factors, including endothelial damage, systemic inflammation, immobility, central venous catheter use, and concurrent immunosuppressive medications.^15^, ^16^, ^17^, ^18^, ^19^, ^20^, ^22^ By contrast, the 3.3% rate in CTCL patients—a population with fewer systemic risk factors for thrombosis—suggests a substantially lower baseline risk when the confounding effects of GVHD‐related comorbidity are removed. If ECP were independently prothrombotic, one would expect a more uniform risk elevation across indications.

The distribution of VTE subtypes in the GVHD cohort is also informative. Upper extremity DVT (2.5%), PE (2.3%), and lower extremity DVT (2.3%) contributed approximately equally. The relatively high proportion of upper extremity events may reflect the prevalent use of central venous catheters in the transplant population, though we note that TriNetX does not reliably capture procedural details such as vascular access type, and this interpretation is therefore speculative.

The low rate of arterial thrombotic events (1.5%–1.7% at 30 days) is notable in light of the Jacobs et al. MAUDE database analysis, which reported transient ischemic attack or cerebrovascular accident in 6 out of the 37 evaluated patients (16%).^6^ This discrepancy likely reflects the difference between passive adverse event reporting, which may enrich for severe or unusual presentations, and population‐level incidence estimation from EHR data.

Taken together, these findings strongly suggest that thrombotic events following ECP are driven primarily by patient‐level factors—particularly the underlying disease and its associated risk profile—rather than by the ECP procedure itself. However, because this is a descriptive study without a matched comparator, a small contributory effect of ECP on thrombotic risk cannot be entirely excluded. Establishing whether such an effect exists would require a controlled study with appropriate adjustment for disease severity and other confounders.

Our findings are consistent with the existing mechanistic literature on ECP and hemostasis. Although in vitro studies have demonstrated platelet activation after 8‐MOP/UVA exposure, this activation does not result in platelet aggregation or measurable changes in soluble thrombotic markers.^10^, ^11^ Coagulation parameters remain stable during long‐term ECP therapy.^12^ The few thrombotic events reported in the published ECP literature have been primarily catheter‐associated, occurring in patients requiring central venous access, rather than systemic.^7^, ^8^, ^9^ The population‐level data presented here reinforce this picture: the absence of periprocedural clustering, the concentration of risk in the GVHD population, and the relatively high proportion of upper extremity events are all more consistent with catheter‐associated and disease‐related thrombosis than with a direct prothrombotic effect of extracorporeal photopheresis.

Our findings have practical implications. The absence of early periprocedural clustering suggests that routine short‐term thromboprophylaxis around individual ECP sessions is unlikely to be beneficial. Rather, clinicians should consider the overall thrombotic risk profile of the patient—particularly those with GVHD—and manage VTE prevention according to established guidelines for the underlying condition. The FDA's recommendation to alert patients and staff to signs and symptoms of PE and DVT during or after ECP procedures remains appropriate but should be contextualized by the understanding that these events likely reflect the patient's baseline risk rather than an acute procedural hazard.

This study has several limitations. First, this is a descriptive study without a comparator group. We did not include a matched cohort of non‐ECP‐treated patients (e.g., GVHD patients not receiving ECP) because the aim was to characterize thrombotic event incidence in ECP‐treated patients, not to determine whether ECP independently increases thrombotic risk. The latter question, while important, would require careful adjustment for disease severity, vascular access, immunosuppressive regimens, and functional status—variables that are not reliably captured in federated EHR data. Future studies using more granular data sources may be better positioned to address this causal question.

Second, as a retrospective analysis of EHR data, we relied on diagnostic codes to identify both ECP procedures and thrombotic outcomes. Coding inaccuracies could result in misclassification in either direction. Third, the subgrouping by indication represents a rough division based on diagnoses that are robustly captured by ICD‐10 coding in the TriNetX platform. It does not constitute a complete classification of all ECP indications; patients receiving ECP for solid organ transplant rejection, for example, are included in the all‐indication cohort but were not analyzed separately. The CTCL cohort was relatively small (*n* = 631, 21 events), and the 3.3% VTE rate (95% CI, 2.1%–5.0%) should be interpreted with appropriate caution given the imprecision inherent in this sample size. Finally, the TriNetX network, while large, may not be fully representative of all ECP practice settings.

## 5 CONCLUSION

VTE following ECP occurs at a rate of approximately 6% at 30 days in the overall ECP population, with a markedly higher rate in patients with GVHD (8.1%; 95% CI, 6.8%–9.5%) compared to CTCL (3.3%; 95% CI, 2.1%–5.0%). Arterial thrombotic events are infrequent (1.5–1.7% at 30 days). These rates fall within the range of published estimates for the underlying patient populations, and the temporal pattern of events—gradual accumulation without periprocedural clustering—strongly suggests that thrombotic risk in ECP‐treated patients is driven by patient‐level factors rather than by the procedure itself, though a small contributory procedural effect cannot be excluded without a controlled comparator study. These findings provide the population‐level denominator that has been absent from prior safety reports and may help clinicians contextualize thrombotic risk in patients receiving ECP.

## FUNDING INFORMATION

No funding was received for this project.

## CONFLICT OF INTEREST STATEMENT

The author has no conflicts of interest to disclose.

## Supporting information


**Supplemental Table S1.** Sensitivity analysis: five‐day risk of venous and arterial thrombosis with and without inclusion of day 0 (all indications).

## Data Availability

The data that support the findings of this study are available from TriNetX. Restrictions apply to the availability of these data, which were used under license for this study. Data are available from https://live.trinetx.com with the permission of TriNetX.
