# Supplementary material for: Venous and arterial thrombosis following extracorporeal photopheresis: A descriptive cohort study using a federated electronic health record network
Source: Transfusion. 2026 Jun 18;66(9):1737–46. doi: 10.1111/trf.70300 (PMC13569314; doi:10.1111/trf.70300)
Supplement: Supplementary file 1 [file TRF-66-1737-s001.docx]

# SUPPLEMENTAL MATERIAL

**Supplemental Table S1.** Sensitivity analysis: five-day risk of venous and arterial thrombosis with and without inclusion of day 0 (all indications)

| **Exclusion Period** | **Outcome** | **Day 1–5 Risk (95% CI)** | **n/N** | **Day 0–5 Risk (95% CI)** | **n/N** | **Survival Prob. (Day 0–5)** |
| --- | --- | --- | --- | --- | --- | --- |
| 6 months | VTE | 1.5% (1.1–2.0) | 46/3,096 | 2.4% (1.9–3.0) | 75/3,096 | 97.57% |
| 6 months | Arterial | 0.4% (0.2–0.7) | 13/3,096 | 0.6% (0.4–1.0) | 20/3,096 | 99.35% |
| 1 year | VTE | 1.4% (1.0–1.8) | 41/3,006 | 2.3% (1.8–2.9) | 70/3,006 | 97.67% |
| 1 year | Arterial | 0.4% (0.2–0.7) | 11/3,006 | 0.6% (0.3–0.9) | 17/3,006 | 99.43% |

*VTE = venous thromboembolism (ICD-10-CM I82 + I26); Arterial = cerebral infarction (I63) or acute myocardial infarction (I21). Day 1–5 = primary analysis (observation window begins the day after the index ECP procedure). Day 0–5 = sensitivity analysis including the day of the index procedure. Events coded on day 0 cannot be reliably determined to have occurred after the ECP procedure due to calendar-day resolution of EHR data. Confidence intervals are exact (Clopper-Pearson) 95% CIs.*
